# Differences in the phospholipid profile of melanocytes and melanoma cells irradiated with UVA and treated with cannabigerol and cannabidiol

**DOI:** 10.1038/s41598-023-43363-9

**Published:** 2023-09-26

**Authors:** Wojciech Łuczaj, Izabela Dobrzyńska, Elżbieta Skrzydlewska

**Affiliations:** 1 Department of Analytical Chemistry, Medical University of Bialystok, Mickiewicza 2d, 15-222 Bialystok, Poland; 2 Faculty of Chemistry, University of Białystok, Ciołkowskiego 1K, 15-245 Białystok, Poland

**Keywords:** Lipidomics, Lipids, Phospholipids

## Abstract

UV radiation inducing mutations in melanocytes might cause melanoma. As changes in lipid composition and metabolism are associated with many types of cancer including skin cancer, we aimed to evaluate the effects of two phytocannabinoids cannabidiol (CBD) and cannabigerol (CBG), on changes in phospholipid and ceramide (CER) profiles induced by UVA irradiation in human melanocytes and melanoma. UVA radiation caused a significant up-regulation PC, PI and SM species and decrease of CERs content in both types of cells, while up-regulation of PEo was only observed in melanocytes. Exposure of UVA-irradiated melanocytes or melanoma cells to CBD and/or CBG led to significant decrease in relative content of PC, PI and SM specie; however, this effect was more pronounced in cancer cells. Interestingly, only in UVA-irradiated melanocytes and not in melanoma, PEo content was lowered after CBD treatment, while CBG led to additional up-regulation of PEo species. CBD and CBG used together caused decrease of zeta potential, inhibiting PS externalization, and different changes in relative contents of CER and SM species of irradiated and non-irradiated melanoma cells. Obtained results are quite promising due to CBD and CBG abilities to partial reverse pro-cancerogenic changes in phospholipid and CER profiles induced by UVA.

## Introduction

One of the most important groups of compounds in skin cells are lipids, which are essential as structural components, but also participate in energy metabolism, cellular signaling, enzyme activation, transmembrane transport as well as cell proliferation, differentiation and apoptosis^1^. The key structural components of cell membranes that play an important role in signal transduction and are also involved in carcinogenesis are phospholipids^2^. Among them phosphatidylcholines and their metabolites have been shown to contribute to both increased proliferation and apoptosis^3^. Lysophosphatidylcholines (LPCs) are considered to be major pro-carcinogenic signaling lipids as elevated levels of these lysophospholipids have been found in various types of cancer^4^. LPCs, by regulating intracellular calcium levels, may indirectly affect the activation of signaling pathways, including p38 MAPK, which increases cell proliferation^2^. These findings allow to claim that the recognition of changes in phospholipid composition is not only valuable source of information on disease progression, but is also indicate specific phospholipids considered as potential biomarkers, that can be used as tool for better diagnosis and prognosis of cancer^5,6^. Therefore alteration in phospholipid metabolism and changes in their composition is hallmark of many types of cancer^7,8^. It has been confirmed that changes in phospholipid profile are correlated with cell differentiation and malignancy^5,9^. Consequently, lipidomics is now one of the main approaches used in cancer research.

It is believed that genetic conditions and exposure to harmful exogenous factors (physical and chemical stressors) including UV radiation, promote structural and functional changes in the components of skin cells, especially epidermal cells, which finally may lead to neoplastic transformation. Exposure to UV radiation is considered the most important physical factor causing mutations in melanocytes. Both UVB and UVA radiation, especially after long-term exposure, have been shown to contribute to the development of melanoma and account for the development of approximately 65% of all types of melanoma^10^. Melanocytes due to their location in deeper part of epidermis are more susceptible to UVA than UVB radiation. Unfortunately, the effect of exposure to UVA radiation on the skin, and especially on its deeper layers, has not been thoroughly studied.

Melanoma is the most aggressive neoplasm with a tendency to metastasize with only 20% rate of 5-year survival^11^. The worldwide incidence of melanoma increased rapidly in recent years being greatest among geriatric populations, although it is also among the most common cancers found in adolescent and young adult populations^12,13^. To date, data on the impact of UV radiation on the development and progression of melanoma are still limited. Nevertheless, it is well established that UVA affects metabolism in tumor cells promoting their uncontrolled growth, survival and resistance to chemotherapy^14^. However, expanding knowledge on complex interactions between UVA and melanocytes, with particular emphasis on the impact of UVA on phospholipid metabolism in terms of melanoma risk or protection against melanomagenesis, is highly desirable. Moreover, despite a significant improvement in cancer treatment strategies in the last decade, the prognosis for patients with melanoma, but particularly with metastatic melanoma is very poor, both due to drug resistance and frequent relapses^15^. Therefore, in order to better understand the processes involved in the progression of melanoma, metabolic changes accompanying the development of this cancer, including changes in lipid composition, are constantly analyzed. In addition, new possibilities of prophylaxis and/or pharmacotherapy are being tested. One of the currently examined approaches is the use of natural compounds with antioxidant/anti-inflammatory properties, especially lipophilic, easily penetrating cell membranes. Among the compounds most often analyzed for this purpose are non-psychoactive phytocannabinoids such as cannabidiol (CBD) and cannabigerol (CBG) with lipophilic properties and the ability to modulate cellular metabolism^16^. Similarly to endocannabinoids, both phytocannabinoids exhibit a wide range of biological activities. These activities include those based on interaction with G-protein-coupled receptors (GPCRs)^17^, which are involved in the regulation of ROS and TNF-α levels, but also play crucial role in melanocyte physiology. GPCRs are also involved in tumorigenesis and metastatic progression of melanoma in all stages. In addition, it has been suggested that regulation of GPCRs may expand the treatment options for melanoma in the future^18^. There is also contradicting evidence on the therapeutic potential of phytocannabinoids for non-melanoma skin cancer and melanoma in vitro and in vivo. The conflicting data observed in non-melanoma skin cancer and melanoma studies may result from various factors affecting cellular metabolism, including dose-dependent effects and that the in vitro studies may not have accounted for the tumor microenvironment^19,20^.

Considering all the above, it seems very important to expand the knowledge about the complex effect of UVA on melanocytes in terms of protection against the potential development of melanoma, based on natural compounds. Thus, the aim of this study was to evaluate the effects of two phytocannabinoids (CBG and CBD), used individually or in combination, on changes in phospholipid and ceramide profiles induced by UVA irradiation in commercially available ATCC cell lines of melanocytes and human melanoma.

## Materials and methods

### Reagents/chemicals

The phospholipid internal standards were purchased from Avanti Polar Lipids, Inc. (Alabaster, AL, USA). All chemicals were purchased from Sigma-Aldrich Chemical Co. (St. Louis, MO, USA); all solvents were of LC–MS grade. Milli-Q water used for all experiments was obtained using a Milli-Q Millipore system by filtering through a 0.22 mm filter (Advantage A10, Millipore Corporation, Billerica, MA, USA).

### Cell culture and treatment

All cell lines used in study were obtained from American Type Culture Collection (ATCC, Manassas, VA, USA). Human melanoma cells (ATCC HTB-70; SK-MEL-5) were isolated from skin tissue of a 24-year-old, white, female malignant melanoma patient, while human primary epidermal melanocytes (ATCC PCS-200-012) were obtained from neonatal foreskin. All cell lines were authenticated by the supplier. Cells were cultured according to ATCC recommendations. SK-MEL-5 cells and melanocytes were cultured in Eagle’s Minimum Essential Medium (EMEM, ATCC-30-2003) and Dermal Cell Basal Medium (DCBM, ATCC-PCS-200-030), respectively, containing fetal bovine serum (10%), epidermal growth factor EGF 1–53 (5 µg/L), 50 U/ml of penicillin, and 50 μg/mL pf streptomycin. The cells were cultured in a humidified atmosphere of 5% CO_2_ at 37 °C. When the cells (passage 3) reached 90% confluence, they were subjected to further treatment.

Next, cells were washed with phosphate-buffered saline (PBS) and in this buffer exposed to UVA (365 nm) at a dose of 20 J/cm^2^ (corresponding to 75 ± 5% cell viability) what was selected basing on the results of MTT assay^21^. The melanocytes and SK-MEL-5 cells were irradiated with UVA in plastic dishes surrounded by ice, 15 cm from the six (6 W) lamps (365 nm) (Bio-Link Crosslinker BLX 312/365; Vilber Lourmat, Germany), corresponding to 4.08 mW/cm^2^. Buffer temperature during exposure do not exceed 8 °C. The total irradiation time was 70 min, during which groups of non-irradiated cells were incubated in identical conditions, in a dark (protected against UVA).

The CBD and CBG treatment was carried out by culturing cells for 24 h (h) in a medium containing 5 µM and 1 µM of CBD and CBG, respectively (Sigma-Aldrich, MO, USA). These concentrations of CBD and CBG did not change the morphology or proliferation of examined cells^22,23^ or the cell viability measured by the MTT assay^21^ (Supplementary Fig. F1).

The melanocytes and SK-MEL-5 cells were divided into eight experimental groups of six samples each:

- Group 1 [Control]: Melanocytes\ SK-MEL-5 cells cultured in standard medium.
- Group 2 [CBD]: Melanocytes\ SK-MEL-5 cells cultured for 24 h in a medium containing 5 µM of CBD.
- Group 3 [CBG]: Melanocytes\ SK-MEL-5 cells cultured for 24 h in a medium containing 1 µM of CBG.
- Group 4 [CBG + CBD]: Melanocytes\ SK-MEL-5 cells cultured for 24 h in a medium containing 5 µM and 1 µM of CBD and CBG, respectively.
- Group 5 [UVA]: Melanocytes\ SK-MEL-5 cells exposed to UVA radiation.
- Group 6 [UVA + CBD]: Melanocytes\ SK-MEL-5 cells exposed to UVA radiation and then cultured for 24 h in a medium containing 5 µM of CBD.
- Group 7 [UVA + CBG]: Melanocytes\ SK-MEL-5 cells exposed to UVA radiation and then cultured for 24 h in a medium containing 1 µM of CBG.
- Group 8 [UVA + CBG + CBD]: Melanocytes\ SK-MEL-5 cells exposed to UVA radiation and then cultured for 24 h in a medium containing 5 µM and 1 µM of CBD and CBG, respectively.

In total the study was conducted on 16 experimental groups – 8 groups of melanocytes and 8 groups of SK-MEL-5 cells as shown on the scheme:
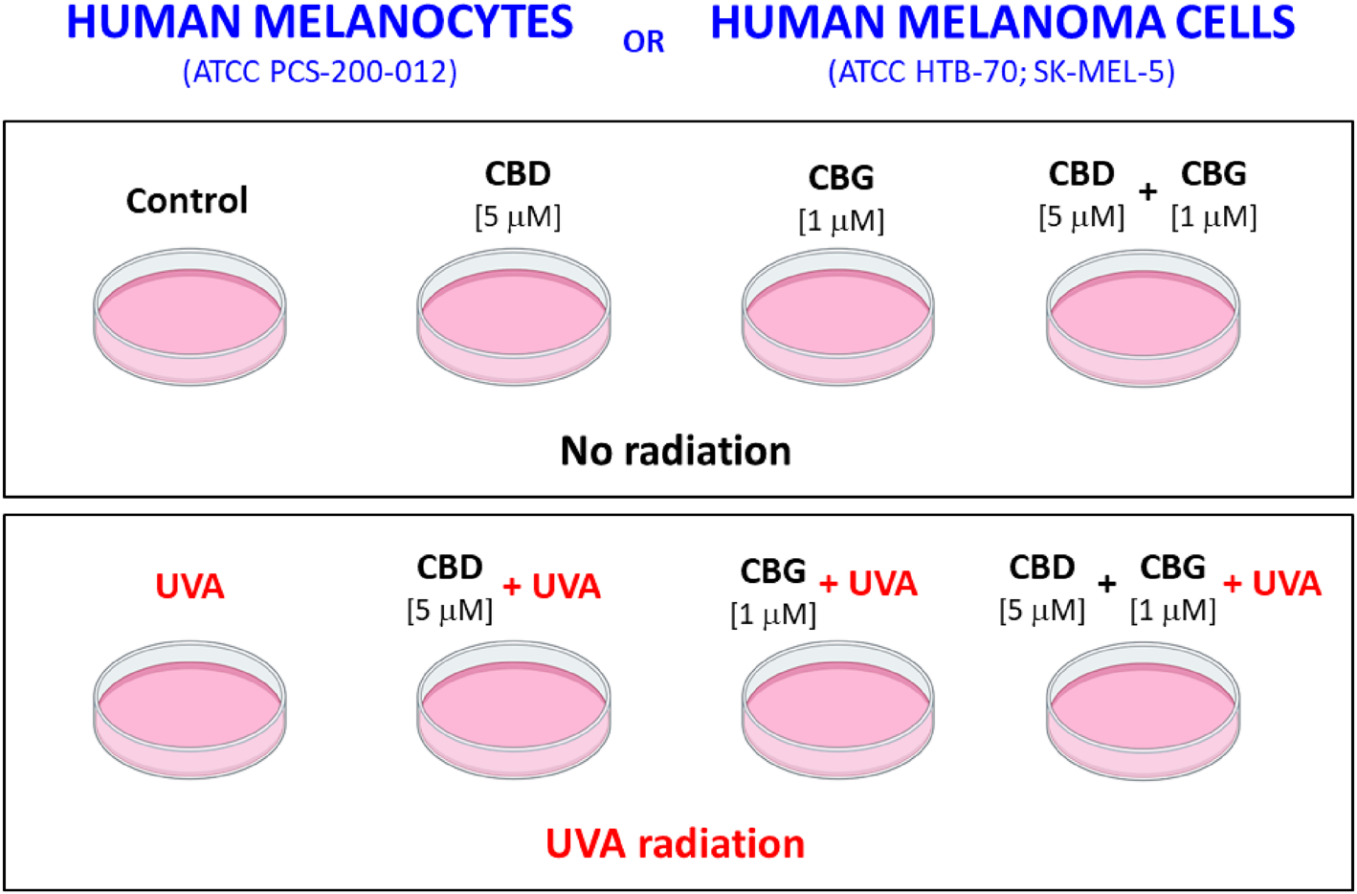


The cells of each group were washed with PBS, collected by scraping into cold PBS and centrifuged.

### Lipid extraction and quantification of total phospholipid content

The Bligh and Dyer method^24^ was used for total lipids extraction from cell pellet. The amount of phospholipids was quantified in each extracts according to the Bartlett and Lewis method^25^. All experimental procedures concerning lipid extraction and phospholipid quantification were described in detail in previously published studies^26,27^.

### Phospholipid profiling by hydrophilic interaction liquid chromatography coupled with high-resolution tandem mass spectrometry (HILIC-MS/MS)

The HILIC-MS/MS was applied to obtained phospholipid profile. UPLC system (Agilent 1290; Agilent Technologies, Santa Clara, CA, USA) coupled with a QTOF mass spectrometer (Agilent 6540; Agilent Technologies, Santa Clara, CA, USA) was applied for analysis. Chromatographic separation of phospholipids was performed on the Ascentis Si column (15 cm × 1 mm, 3 μm, Sigma-Aldrich) in gradient elution with the mixture of solvent A [ACN/MeOH/water 50:25:25 (v/v/v) with 1 mM ammonium acetate] and solvent B [ACN/MeOH 60:40 (v/v) with 1 mM ammonium acetate]. The QTOF mass spectrometer operated in negative-ion mode (electrospray voltage, − 3000 V) with capillary temperature, 250 °C and sheath gas flow of 13 L/min, as previously described in details^28^.

### Ceramide profiling by reversed-phase chromatography coupled with high-resolution tandem mass spectrometry RPLC-MS/MS analysis of ceramides

Ceremide profiles were obtained by using the same Agilent UPLC-ESI-QTOF-MS system as in the case of phospholipid profiling (Agilent 1290; Agilent 6540; Agilent Technologies, Santa Clara, CA, USA). Ceremides were separated by RPLC on the RP C18 column (Acquity BEH Shield 2.1 × 100 mm; 1.7 μm; Waters, Milford, MA, USA) using methanol and water with 20 mM ammonium formate pH 5. The QTOF operating parameters and identification of ceramide species was previously described in detail^29^.

### Data processing

Data processing including filtering, peak detection, alignment, integration and the assignment of each phospholipid and ceramide species was performed with use of MZmine 2.30 software based on in-house lipid database^30^. Phospholipid and ceramide species were confirmed by mass accuracy typically less than 5 ppm (Supplementary Table S1 and S2).

### Statistical analysis

Metaboanalyst version 4.0^31^ was applied for univariate and multivariate statistical analyses. Principal component analysis (PCA) was performed on autoscaled data obtained by MS/MS analysis. Additional statistically significant differences between both cell lines submitted to different treatments were investigated using one-way ANOVA test with Tukey’s post hoc test. A P < 0.05 was considered as statistically significant. The heatmaps were created using "Euclidean" as the clustering distance and "Ward" as the clustering algorithm.

### Physicochemical properties of cell membranes; analysis of the zeta potential

The cells were suspended in 0.9% NaCl and placed in a measuring vessel. The zeta potential of cell membranes was measured using a Zetasizer Nano ZS apparatus (Malvern Instruments, UK).

### Statistical analysis

The data are expressed as average ± SD (for n = 6). The data were analyzed using One way ANOVA with the Scheffe's F test for multiple comparisons to determine the significance of the differences between groups. A P value < 0.05 was considered significant. Statistical analyses were performed using *SPSS software* (IBM Japan v.20.0, Japan).

## Results

Using the lipidomic approach based on the hydrophilic interaction liquid chromatography coupled to electrospray ionization high resolution tandem mass spectrometry (HILIC-LC–MS/MS), we identified seven phospholipid classes in the obtained cell extracts: phosphatidylethanolamine (PE), lyso-phosphatidylethanolamine (LPE), phosphatidylcholine (PC), lyso-phosphatidylcholine (LPC), phosphatidylserine (PS), sphingomyelin (SM), and phosphatidylinositol (PI). We recognized 113 and 104 most abundant phospholipid species in the melanocytes and SK-MEL-5cells, respectively (supplementary Table S1 and Table S2). The relative quantification of these phospholipid species was performed based on The peak areas of each phospholipid specie listed in supplementary Tables S3 and Table S4, were used for relative quantification as previously described^28^.

We applied multivariate and univariate statistics to investigate significant changes between phospholipid profiles of examined groups. We analyzed two data sets corresponding to both cell lines (melanocytes and SK-MEL-5cells) each comprising four groups: control (normal melanocytes and SK-MEL-5 cells), cells irradiated with UVA (UVA) and cells irradiated with UVA and treated with cannabidiol (5 µM) (CBD), cannabigerol (1 µM) (CBG) and in combination (CBD + CBG).

**Figure 1:**
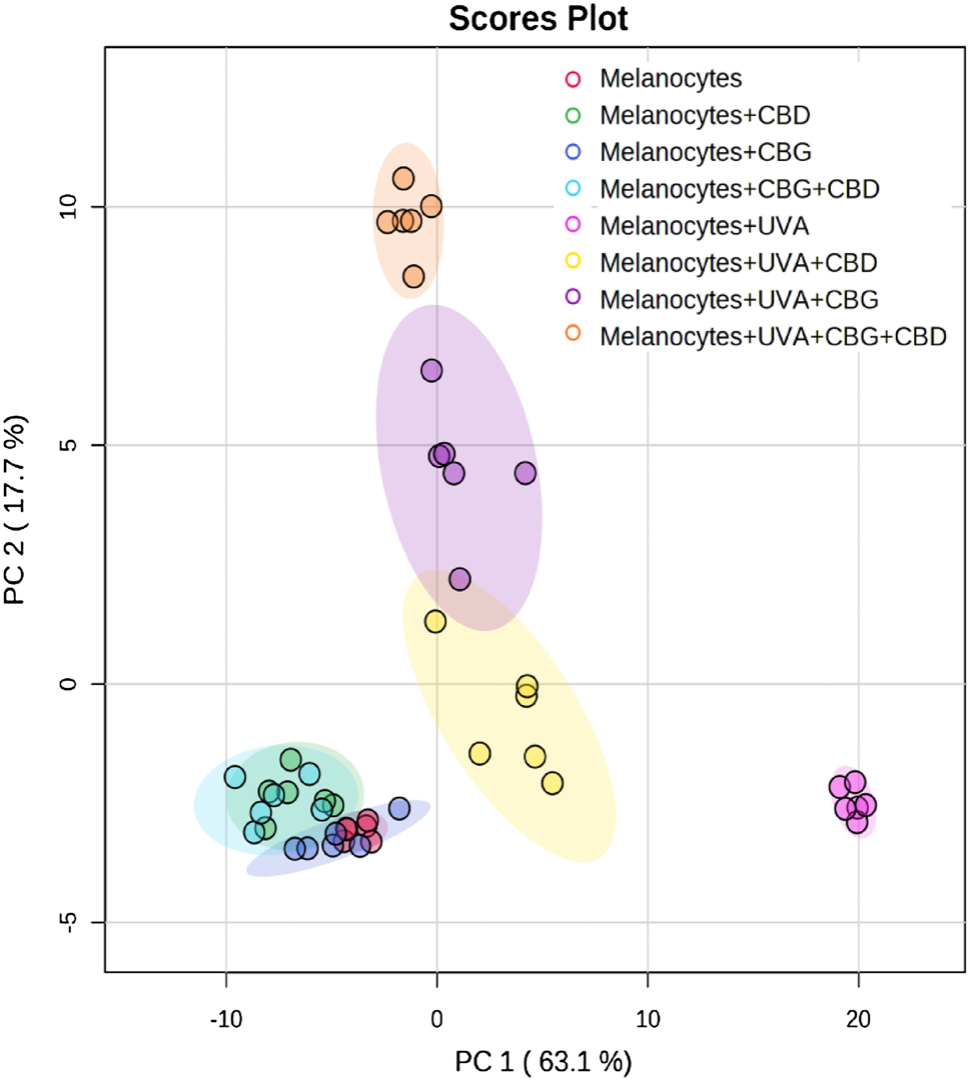
Two-dimensional principal component analysis (2D PCA) scores plot of the relative phospholipid content in non-irradiated (melanocytes), irradiated with UVA (melanocytes + UVA) and treated with cannabidiol (5 µM) (melanocytes + CBD), cannabigerol (1 µM) (melanocytes + CBG) and in combination (melanocytes + CBG + CBD).

The PCA of phospholipid species in melanocytes explained 80.8% of the variance (PC1: 63.1%, PC2: 17.7%) (Fig. 1). The difference between analyzed groups, in particular with regard to the group of UVA-irradiated melanocytes, in multivariate space lined up most with the PC1 component. PC2 component mainly describes the variation within the groups, in respect to the groups of UVA-irradiated melanocytes and treated with CBG or treated with both CBG and CBD.

The PCA plot shows complete separation of UVA-irradiated melanocytes from the other seven analysed groups of cells (Fig. 1). Three groups of UVA-irradiated melanocytes (namely, Melanocytes + UVA + CBD, Melanocytes + UVA + CBG and Melanocytes + UVA + CBG + CBD) were also well separated from four groups of non-irradiated cells (Melanocytes, Melanocytes + CBD, Melanocytes + CBG and Melanocytes + CBG + CBD), which were very poorly separated from each other.

**Figure 2:**
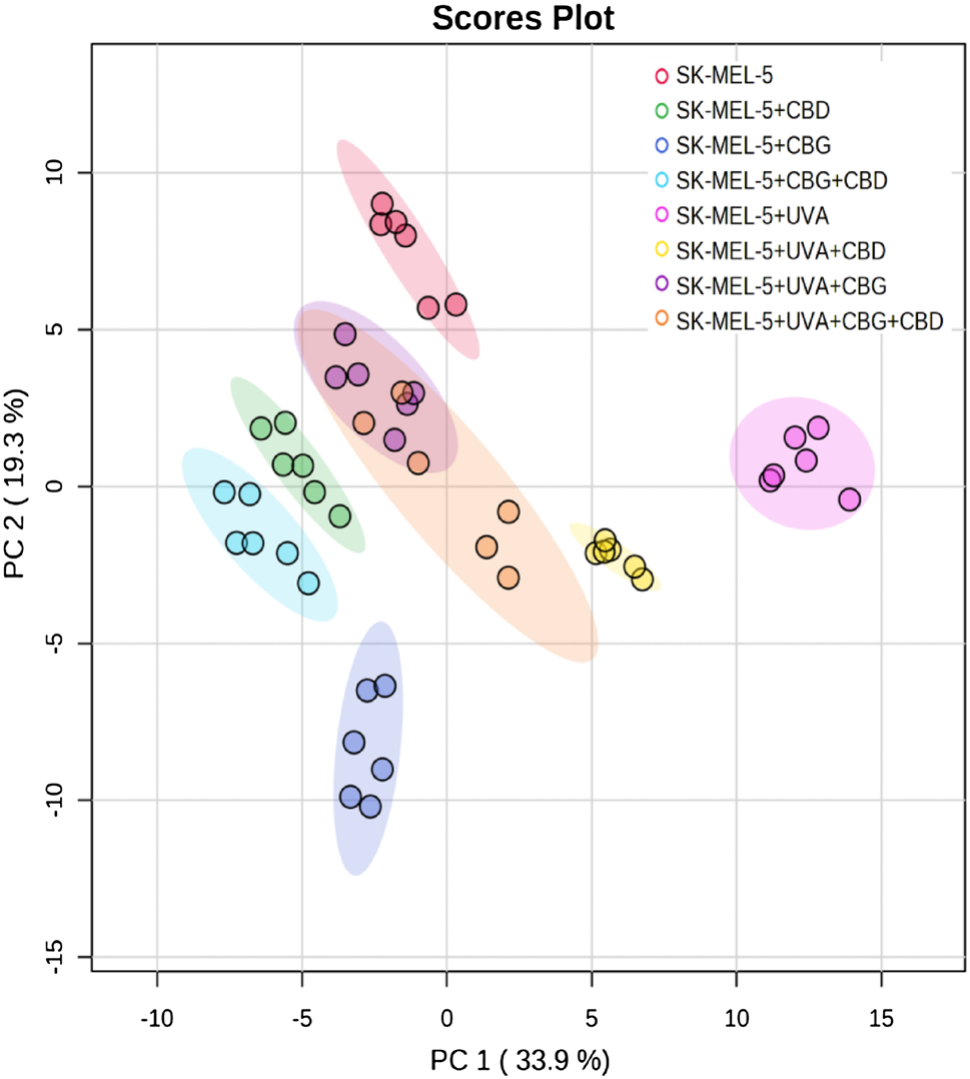
Two-dimensional principal component analysis (2D PCA) scores plot of the relative phospholipid content in SK-MEL-5 cells, non-irradiated SK-MEL-5 cells (SK-MEL-5) and irradiated with UVA (SK-MEL-5 + UVA) and treated with cannabidiol (5 µM) (SK-MEL-5 + CBD), cannabigerol (1 µM) (SK-MEL-5 + CBG) and in combination (SK-MEL-5 + CBG + CBD).

The PCA of phospholipid variables in SK-MEL-5 cells explained 53.2% of the variance (PC1: 33.9%, PC2: 19.3%) (Fig. 2). The differences between analyzed groups lie on a diagonal line in the two-dimensional PCA model. It should be noted that all examined groups of cancer cells were very well separated from each other with the exception of SK-MEL-5 + UVA + CBG and SK-MEL-5 + UVA + CBG + CBD groups (Fig. 2). However, PC1 component mainly describes the variation between both SK-MEL-5 + UVA and SK-MEL-5 + UVA + CBD groups and the other analysed groups of cancer cells.

For a more thorough analysis of the data obtained, we performed a one-way ANOVA and Tukey's post hoc test for melanocytes and SK-MEL-5 cells (Tables 1 and 2).

**Table 1:** The alteration observed in the molecular species of 25 most discriminating (according to one-way ANOVA) phospholipid species from PEo, PC, PI and SM class in non-irradiated (melanocytes), irradiated with UVA (melanocytes + UVA) and treated with cannabidiol [CBD (5 µM)], cannabigerol [CBG (1 µM)] or both phytocannabinoids (CBG + CBD).

Melanocytes						
PL class	Phospholipid specie	Phospholipid molecular specie	Fold-change			
			UVA vs control	UVA + CBD vs UVA	UVA + CBG vs UVA	UVA + CBGCBG + CBD vs UVA
PE	PEo(36:1)	PE(O-18:0/18:1)	1.06 ↑	0.38 ↓	1.46 ↑	2.05 ↑
PE	PEo(34:1)	PE(O-16:0/18:1)	0.92 ↑	0.52 ↓	0.69 ↑	1.27 ↑
PE	PEo(34:2)	PE(O-16:0/18:2)	0.93 ↑	0.63 ↓	0.19 ↑	0.69 ↑
PE	PEo(34:3)	PE(O-16:0/18:3)	0.99 ↑	0.55 ↓	0.30 ↑	0.88 ↑
PE	PEo(36:3)	PE(O-16:0/20:3)	0.93 ↑	0.46 ↓	0.49 ↑	1.06 ↑
PE	PEo(40:4)	PE(O-18:0/22:4)	0.89 ↑	0.42 ↓	0.22 ↑	0.79 ↑
PE	PEo(38:4)	PE(O-16:0/22:4)	1.05 ↑	0.48 ↓	0.16 ↑	0.63 ↑
PE	PEo(38:6)	PE(O-16:0/22:6)	0.95 ↑	0.98 ↓	0.71 ↑	0.12 ↓
PE	PEo(36:5)	PE(O-16:0/20:5)	1.01 ↑	0.75 ↓	0.57 ↑	0.10 ↑
PE	PEo(40:5)	PE(O-18:0/22:5)	0.97 ↑	0.70 ↓	0.40 ↑	0.19 ↑
SM	SM(d42:2)	SM(d18:1/24:1)	1.43 ↑	0.85 ↓	0.66 ↓	1.06 ↓
PC	PC(34:0)	PC(16:0/18:0)	1.40 ↑	0.49 ↓	0.88 ↓	1.14 ↓
PC	PC(32:1)	PC(16:0/16:1)	1.46 ↑	0.59 ↓	0.97 ↓	1.36 ↓
PC	PC(40:4)	PC(18:0/22:4)	1.38 ↑	0.59 ↓	0.98 ↓	1.13 ↓
PC	PC(42:2)	PC(24:0/18:2)	1.34 ↑	0.54 ↓	0.92 ↓	1.16 ↓
PC	PC(38:4)	PC(16:0/22:4)	1.27 ↑	0.99 ↓	1.32 ↓	1.54 ↓
PC	PC(38:6)	PC(16:0/22:6)	1.17 ↑	0.30 ↓	0.68 ↓	1.11 ↓
SM	SM(d42:3)	SM(d18:2/24:1)	1.40 ↑	0.80 ↓	1.51 ↓	1.96 ↓
PI	PI(40:7)	PI(18:1/22:6)	2.08 ↑	1.19 ↓	0.44 ↓	0.71 ↓
PC	PC(36:4)	PC(16:0/20:4)	1.48 ↑	1.07 ↓	1.45 ↓	0.92 ↓
PC	PC(40:6)	PC(18:0/22:6)	1.21 ↑	0.77 ↓	1.16 ↓	1.38 ↓
PI	PI(38:4)	PI(18:0/20:4)	2.10 ↑	1.66 ↓	2.61 ↓	0.98 ↓
PI	PI(40:4)	PI(18:0/22:4)	2.06 ↑	1.73 ↓	1.89 ↓	1.59 ↓
PI	PI(40:6)	PI(18:0/22:6)	1.99 ↑	2.21 ↓	0.91 ↓	2.67 ↓
PI	PI(36:4)	PI(16:0/20:4)	2.05 ↑	2.28 ↓	2.44 ↓	2.77 ↓

**Table 2:** The alteration observed in the molecular species of 25 most discriminating (according to One-way ANOVA) phospholipid species from PEo, PC, PI and SM class in non-irradiated SK-MEL-5 cells (SK-MEL-5), irradiated with UVA (SK-MEL-5 + UVA) and treated with cannabidiol [CBD (5 µM)], cannabigerol [CBG (1 µM)] or both phytocannabinoids (CBG + CBD).

SK-MEL-5 cells						
PL class	Phospholipid specie	Phospholipid molecular specie	Fold-change			
			UVA vs control	UVA + CBD vs UVA	UVA + CBG vs UVA	UVA + CBG + CBD vs UVA
PC	PC(34:0)	PC(16:0/18:0)	0.39 ↑	1.27 ↓	3.01 ↓	1.87 ↓
PC	PC(38:5)	PC(16:0/22:5)	1.24 ↑	1.64 ↓	3.05 ↓	2.10 ↓
PC	PC(38:6)	PC(16:0/22:6)	1.73 ↑	1.71 ↓	3.93 ↓	3.16 ↓
PC	PC(32:1)	PC(16:0/16:1)	1.79 ↑	1.57 ↓	2.81 ↓	2.82 ↓
PC	PC(38:2)	PC(18:0/20:2)	2.20 ↑	2.04 ↓	3.62 ↓	3.62 ↓
PC	PC(40:4)	PC(18:0/22:4)	1.72 ↑	2.18 ↓	3.85 ↓	3.53 ↓
PC	PC(40:6)	PC(18:0/22:6)	1.19 ↑	1.29 ↓	3.96 ↓	4.05 ↓
PC	PC(40:2)	PC(18:0/22:2)	1.05 ↑	1.25 ↓	3.76 ↓	4.07 ↓
PC	PC(40:5)	PC(18:0/22:5)	0.89 ↑	1.31 ↓	4.03 ↓	3.47 ↓
PC	PC(32:0)	PC(16:0/16:0)	0.33 ↑	1.33 ↓	3.10 ↓	2.74 ↓
PC	PC(38:3)	PC(18:0/20:3)	1.87 ↑	1.61 ↓	4.72 ↓	4.48 ↓
PC	PC(36:3)	PC(16:0/20:3)	1.38 ↑	1.35 ↓	3.30 ↓	3.14 ↓
PI	PI(38:6)	PI(16:0/22:6)	8.36 ↑	7.11 ↓	13.60 ↓	6.77 ↓
PI	PI(36:2)	PI(18:0/18:2)	7.26 ↑	2.73 ↓	6.58 ↓	6.92 ↓
PI	PI(40:5)	PI(18:0/22:5)	7.17 ↑	0.10 ↑	5.53 ↓	6.25 ↓
PI	PI(36:0)	PI(18:0/18:0)	8.71 ↑	6.27 ↓	8.18 ↓	7.88 ↓
PI	PI(38:2)	PI(18:0/20:2)	7.29 ↑	4.91 ↓	6.29 ↓	6.63 ↓
PI	PI(38:7)	PI(16:1/22:6)	8.32 ↑	6.11 ↓	7.23 ↓	6.07 ↓
PI	PI(38:3)	PI(18:0/20:3)	8.01 ↑	5.12 ↓	6.61 ↓	5.32 ↓
PI	PI(36:3)	PI(18:0/18:3)	7.43 ↑	3.62 ↓	5.05 ↓	4.03 ↓
PI	PI(40:6)	PI(18:0/22:6)	7.67 ↑	4.56 ↓	5.98 ↓	5.88 ↓
SM	SM(d42:3)	SM(d18:2/24:1)	7.23 ↑	3.11 ↓	3.11 ↓	3.57 ↓
PI	PI(34:2)	PI(16:0/18:2)	7.57 ↑	8.81 ↓	7.11 ↓	7.70 ↓
PI	PI(36:4)	PI(16:0/20:4)	7.01 ↑	9.50 ↓	7.20 ↓	8.37 ↓
PI	PI(38:5)	PI(18:1/20:4)	8.02 ↑	9.39 ↓	6.88 ↓	7.67 ↓

**Figure 3:**
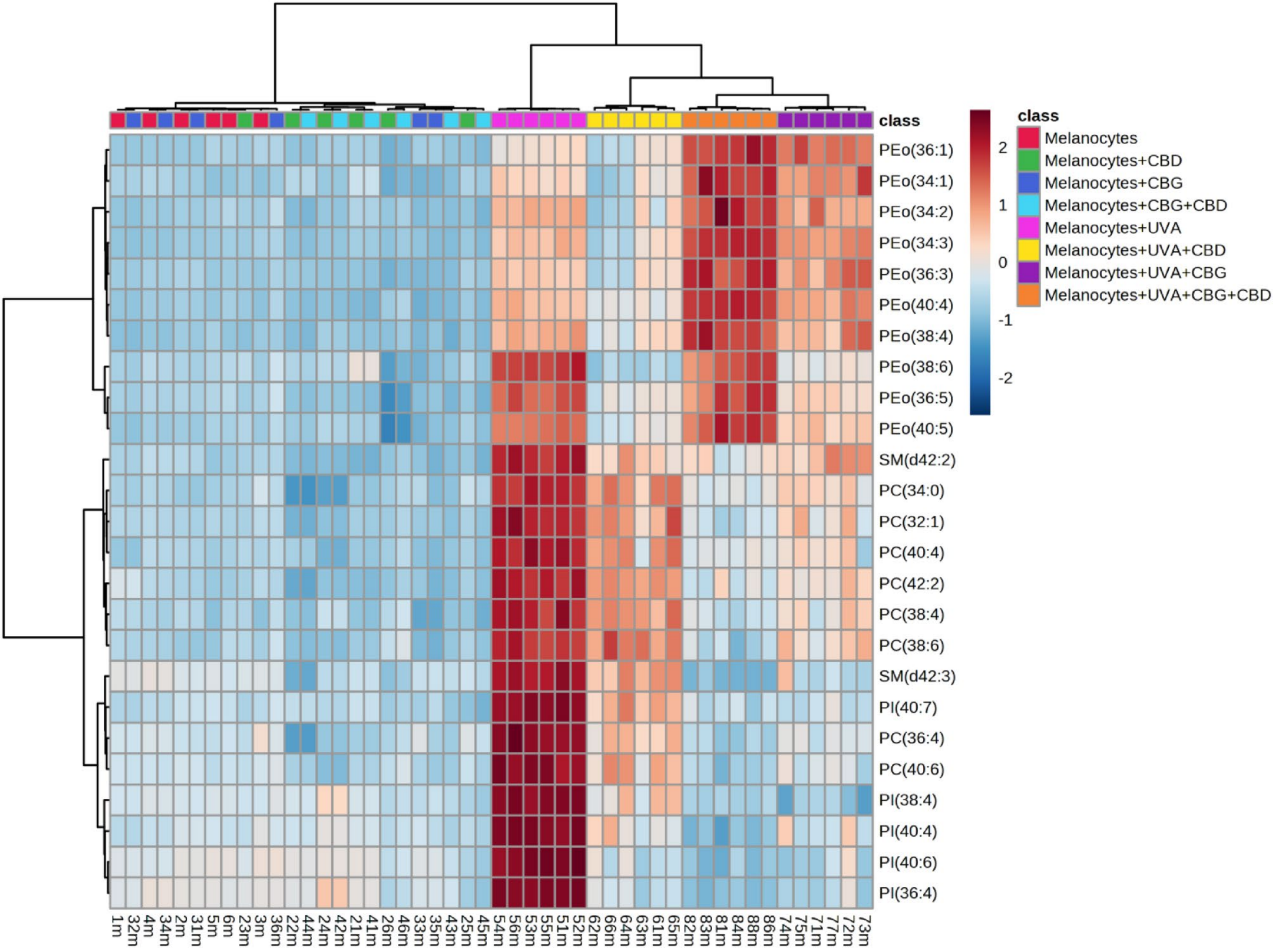
Two-dimensional hierarchical clustering heat map of the 25 main phospholipids of the eight groups of melanocytes, non-irradiated (melanocytes), irradiated with UVA (melanocytes + UVA) and treated with cannabidiol (5 µM) (CBD), cannabigerol (1 µM) (CBG) or both phytocannabinoids (CBG + CBD), after the one-way ANOVA test.

**Figure 4:**
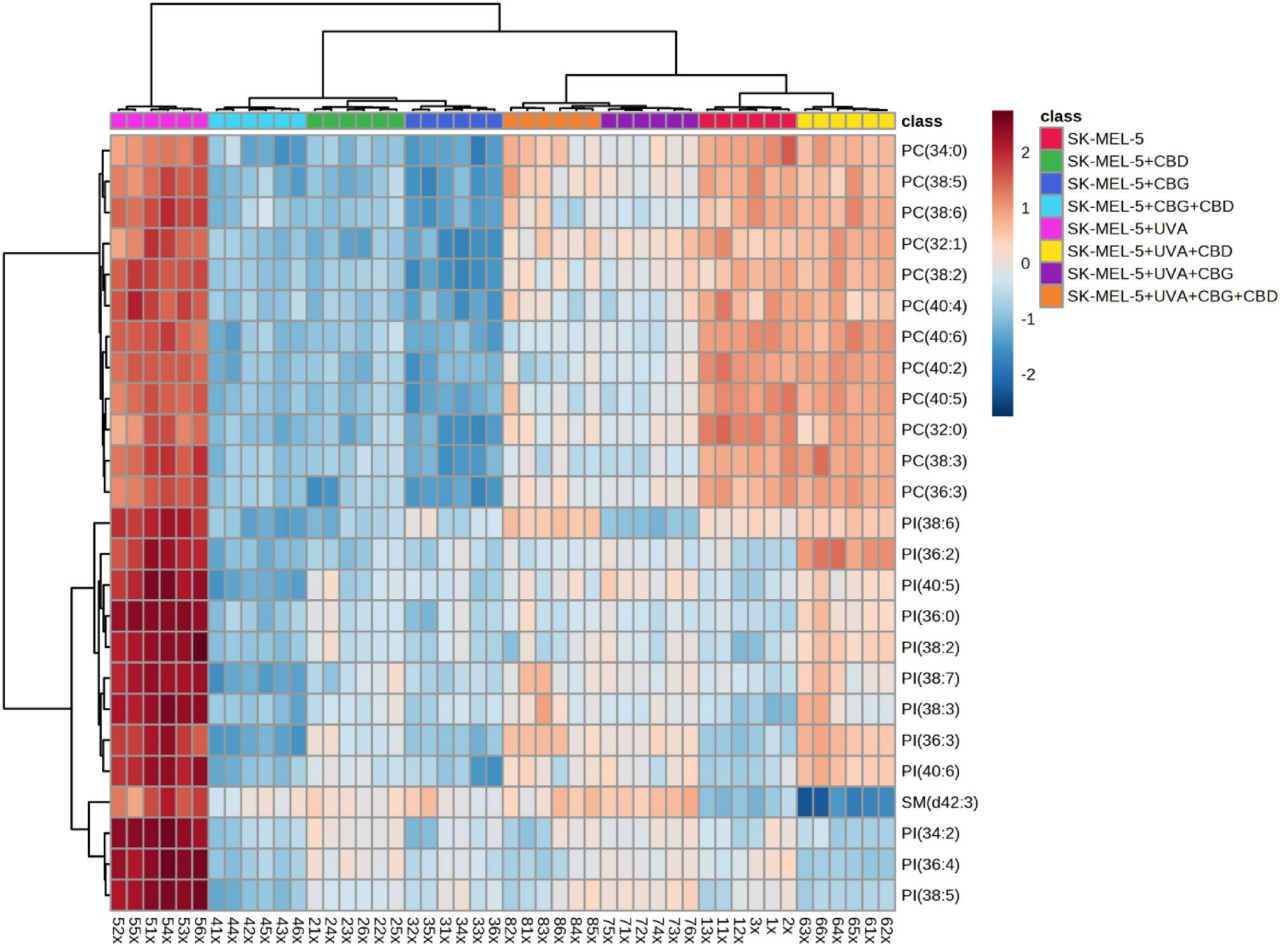
Two-dimensional hierarchical clustering heat map of the 25 main phospholipids of the eight groups of SK-MEL-5 cells, non-irradiated (SK-MEL-5) and irradiated with UVA (SK-MEL-5 + UVA) and treated with cannabidiol (5 µM) (SK-MEL-5 + CBD), cannabigerol (1 µM) (SK-MEL-5 + CBG) or both phytocannabinoids (SK-MEL-5 + CBG + CBD), after the one-way ANOVA test.

The variation in relative abundance of the 25 the most relevant phospholipid species within each class under the conditions studied is presented on dendrograms with two-dimensional hierarchical clustering created for both types of cells melanocytes (Fig. 3) and SK-MEL-5 cells (Fig. 4). The samples were clustered in five (Fig. 3) and four (Figue 4) main groups of samples, in analysis of melanocytes and SK-MEL-5 cells, respectively. However, the individual phospholipid species were clustered into two main groups, in both types of cells. In the case of melanocytes the first group was mainly composed of PE species, while the second group consisted of PI, PC species and two SM species, namely SM(d42:2) and SM(d42:3) (Fig. 3).

In the set of SK-MEL-5 cells, the first group of clustered phospholipids included PC species only, while the second cluster was formed by PI species and SM(d42:3) (Fig. 4).

**Figure 5:**
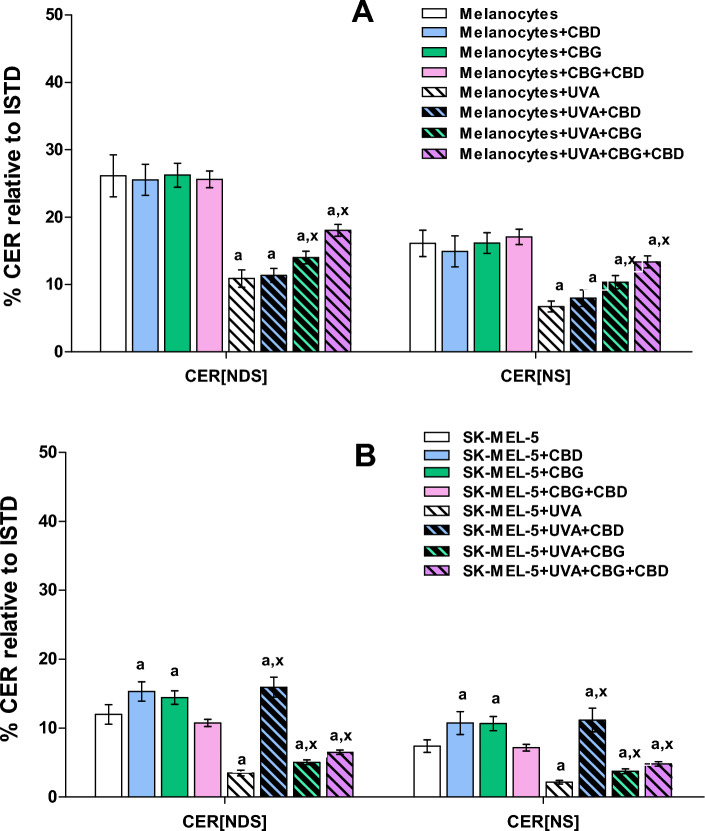
Changes in relative ceramide content within CER[NDS] and CER[NS] classes in melanocytes (**A**) and SK-MEL-5 cells (**B**) non-irradiated and irradiated with UVA (UVA) and treated with cannabidiol (5 µM) (CBD), cannabigerol (1 µM) (CBG) and in combination (CBG + CBD). Values are mean ± SD, *p* < 0.05; a, significantly different melanocytes/SK-MEL-5 cells; x, significantly different from UVA.

We also evaluated alterations in the ceramide profile of examined cells by RPLC-QTOF-MS/MS. Obtained results showed that UVA radiation caused alterations in phospholipid profile of both types of irradiated cells melanocytes and SK-MEL-5. We found that exposure of both melanocytes and SK-MEL-5 cells to UVA led to significant up-regulation of PE, PC, PI and SM species (Figs. 3 and 4, Tables 1, 2), while abundances of CER[NS] and CER[NDS] were significantly decreased (Fig. 5A, B). We found that UVA-irradiation of SK-MEL-5 cells led also to down-regulation of CERs (Fig. 5B).

No significant changes have been observed in the phospholipid profile of melanocytes exposed to CBD and CBG action separately and in combination, while CERs content was significantly elevated after exposed to CBD or CBG action (Figs. 3, 5A, Table 1). In contrast to melanocytes, treatment of SK-MEL-5 cells with CBD and/or CBG led to significant decrease of relative content of PI and PC species, while significant increase of CER[NS] and CER[NDS] was observed only when phytocannabinoids were used separately (Figs. 4 and 5B). In addition, most significant decrease of PI content was observed in SK-MEL-5 cells when CBD and CBG were used in combination, while decrease of PC species content was most pronounced in SK-MEL-5 cells treated only with CBD (Fig. 4, Table 2). Surprisingly, no significant changes in CERs content were observed in melanocytes and SK-MEL-5 cells after treatment with both CBD and CBG (Fig. 5).

However, we found that exposure of UVA-irradiated melanocytes to action of CBD and/or CBG partially reversed changes in phospholipid profile caused by UVA, namely led to significant decrease in relative content of PC, PI and SM species (Fig. 3, Table 1). This effect was most significant in UVA-irradiated melanocytes after treatment with both CBD and CBG. Similarly, treatment of UVA-irradiated SK-MEL-5 cells with CBD and/or CBG led to significant decrease of PI and PC content, particularly when cells were exposed to action of CBG action or both CBG and CBD (Fig. 4, Table 2). Moreover treatment of SK-MEL-5 cells with CBG and/or CBD resulted in significant lowering of SM(d42:3) (Fig. 4, Table 2) and corresponding significant increase of CERs content in this group of SK-MEL-5 cells (Fig. 5). Interestingly most dramatic decrease in content of this SM specie was observed in group of SK-MEL-5 cells treated with CBD (Fig. 4, Table 2), that was accompanied by dramatically elevated CERs content in this group of SK-MEL-5 cells (Fig. 5). However, we also found that in contrast to UVA-irradiated SK-MEL-5 cells, PE was another phospholipid class, which composition changed in UVA-irradiated melanocytes after treatment with phytocannabinoids examined in this study. Relative content of PE species was lowered in UVA-irradiated melanocytes after CBD treatment, while exposure of these cells to CBG led to significant up-regulation of ether linked PE molecular species (PEo), especially when CBG was combined together with CBD (Fig. 3, Table 1).

**Figure 6:**
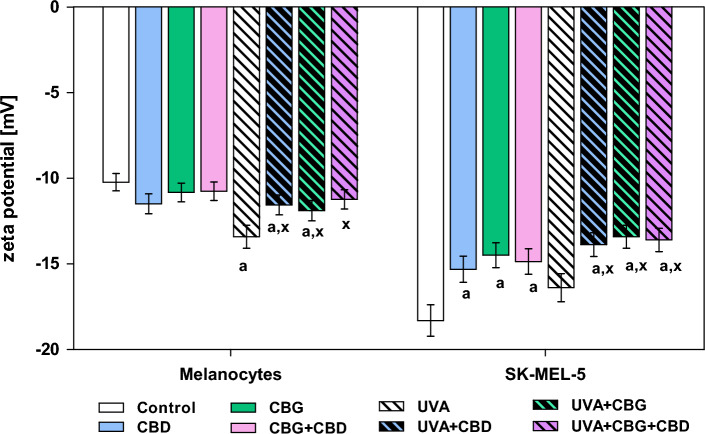
The changes in the Zeta potential of melanocytes and SK-MEL-5 cells non-irradiated and irradiated with UVA (UVA) and treated with cannabidiol (5 µM) (CBD), cannabigerol (1 µM) (CBG) or with both phytocannabinoids (CBG + CBD). Values are mean ± SD, *p* < 0.05; a, significantly different melanocytes/SK-MEL-5 cells; x, significantly different from UVA.

In the cell membranes of SK-MEL-5, an increase in the negative zeta potential was observed estimated at 80% compared to melanocytes (Fig. 6).

UVA radiation caused a significant increase in zeta potential only in the case of melanocytes compared to control cells, while no significant changes were observed in SK-MEL-5 cells after UVA irradiation. It was found that treatment of UVA-irradiated melanocytes with CBD or CBG significantly prevented UVA-induced changes in zeta potential. Importantly, both phytocannabinoids used in combination restored values of zeta potential similar to those of control melanocytes. Significant decrease in negative zeta potential was observed in both non-irradiated and UVA-irradiated SK-MEL-5 cells treated with CBD/CBG/CBG + CBD.

## Discussion

In addition to the fact that lipids are the main components of cell membranes, they also act as signaling molecules and an energy reservoir for cells^32^. Thus, altering their metabolism plays a key role both in cancer development, including tumor cells proliferation, and is important for cancer progression and metastasis^6^. Among phospholipids, phosphatidylcholine (PC) and phosphatidylethanolamine (PE) species represent major components, increased levels of which were observed in melanoma^33^. Results of this study also confirm significant up-regulation of PC as well as some phosphatidylinositol (PI) species in SK-MEL-5 cells compared to melanocytes. It has also previously been shown that the development and progression of melanoma are accompanied by an increase in the level of PI species^34^, the metabolism of which leads to the formation of phosphatidylinositol-3,4,5-trisphosphate (PIP3) via the PI3K/AKT pathway. It should be stressed that PI are also a precursor of phosphoinositides (PIPs), being important for cell signaling^35^. It was shown that the inhibition of PI3K can cause PI increase associated with chemoresistance in human cancer cells^36^. However, higher expression of PIP3 was found in metastatic melanoma compared to primary melanoma cells, resulting in the more invasive phenotype of melanoma cells^37^, while increased expression of AKT was associated with a poor five-year melanoma-patient survival rate^38^. Moreover, PIP3 can be dephosphorylated by the lipid-phosphate phosphatase and tensin homolog deleted on chromosome ten (PTEN) into phosphatidylinositol 4,5-bisphosphate (PIP2), but genetic inactivation of PTEN is frequently found in melanoma^39^, limiting this transformation.

UV is one of the basic environmental factors inducing mutations in melanocytes, hence it is highly probable that UV radiation may contribute to melanomagenesis^40^. Up to now effects of UVA radiation on melanocytes and melanoma cells has not been widely studied. The results of this study showed that UVA radiation causes changes in the phospholipid profile of both normal and cancer cells, melanocytes and SK-MEL-5 cells, namely a significant upregulation of PE, PC, PI and SM species, accompanied by a decrease in the content of CER[NS] and CER[NDS]. As significant up-regulation of PC and PI species has been previously shown in melanoma cells^33,34^ and is observed in this study, it can be speculated that the increase in PI and PC contents in UVA-irradiated melanocytes observed in this study indicates changes that may may suggest the involvement of UVA in melanogenesis and pro-cancerogenic effects. In addition, since PI species content was reported to increase with cancer’s stage in murine^41^ and human melanoma^34^, observed in this study additional increase of PI content in UVA-irradiated both melanocytes as well as SK-MEL-5 cells may indicate an increased susceptibility of melanocytes to malignant transformation under the influence of UVA, as well as increased metastatic potential of melanoma, resulting in further cancer progression.

Due to daily exposure to UVA-containing solar radiation, there is a constant search for substances/compounds, especially of natural origin, that could protect the skin against the potential development of melanoma. Therefore two non-psychoactive phytocannabinoids (CBG and CBD) have been extensively studied in the last years due to their anti-inflammatory and antioxidant properties^42–45^. Moreover, the effects of CBD on phospholipid and ceramide profiles of UV-irradiated skin cells (keratinocytes and fibroblasts) have been also reported^26,46^. Therefore, in this study, we also assessed the effects of both phytocannabinoids on changes in phospholipid and ceramide profiles of melanocytes and SK-MEL-5 cells induced by UVA. No significant changes have been observed in the phospholipid profile and CERs (CER[NS] and CER[NDS]) contents in melanocytes exposed to CBD and CBG action separately and in combination. However, results obtained in this study also revealed that exposure of UVA-irradiated melanocytes to action of CBD and/or CBG partially reversed changes in phospholipid profile caused by UVA, namely led to significant decrease in relative content of PC, PI and SM species. This effect was most significant in UVA-irradiated melanocytes after treatment with both CBD and CBG. Detailed analysis of the phosphatidyloethanolamine (PE) class in UVA irradiated melanocytes showed specific responses of these cells after their treatment with CBD and/or CBG. Relative content of ether linked PE molecular species (PEo) was lowered in UVA-irradiated melanocytes after CBD treatment, while exposure of these cells to CBG led to significant up-regulation of PEo, especially when CBG was used in combination with CBD. The observed significant up-regulation of PEo can be associated with a response to inflammatory processes induced by the exposure of melanocytes to UVA radiation, which is consistent with the results of other studies showing that CBD and CBG have a clear anti-inflammatory effect, especially in the case of counteracting the effects of UV radiation^47–49^.

In contrast to melanocytes, treatment of SK-MEL-5 cells with CBD and/or CBG led to significant decrease in relative content of PI and PC species. Thus, we may assume that examined phytocannabinoids rather act in pathological condition by inducing changes in phospholipid and ceramides composition of melanoma cells than normal epidermal cells, melanocytes. It should be noted, that decrease of PC species was most pronounced in SK-MEL-5 cells treated only with CBD, while most significant decrease of PI content was observed in SK-MEL-5 cells when CBD and CBG were used in combination, what may indicate on synergistic action of both phytocannbinoids. PC species have been shown to contribute to both proliferation and programmed cell death^50^, while higher levels of PI and alterations in its profile have been observed in breast cancer cells^51,52^. Therefore, the observed decrease of PC and PI relative content in UVA-irradiated SK-MEL-5 cells, particularly when these cells were exposed to action of CBG action or both CBG and CBD, can be perhaps an important signal indicating inhibition of melanoma progression.

Taking into account cancer pharmacotherapy, upregulation of CER synthesis seems important, because CERs are considered pro-apoptotic factors. On the other hand, a reduced level of CERs may indicate their increased metabolism, leading to the formation of glycosphingolipids, including gangliosides, which play an important role in cancer progression^53^. In addition, it has been shown that dysregulation of sphingolipid metabolism in melanoma, especially the sphingomyelin-ceramide pathway, protects melanoma cells from death by lowering the level of CERs involved in promoting apoptosis^54^. Furthermore, it has been evidenced that gene encoding sphingomyelin synthase 1 (SMS1), an enzyme which triggers the ceramide conversion to SM, was down-regulated in melanoma^55^. SMS1 down-regulation was also frequently associated with a bad prognosis in metastatic melanoma patients^55^. Moreover, the expression of the acid sphingomyelinase (A-SMase), which hydrolyzes SM into CER, was negatively correlated with melanoma aggressiveness^56^. In this study we found that treatment of SK-MEL-5 cells with CBD or CBG led to significant increase in relative content of CER[NS] and CER[NDS]. This effect is very important suggesting stimulation of ceramides synthesis in UV-irradiated SK-MEL-5 cells resulted from phytocannabinoids treatment. Importantly, our results indicate on CBD, as compound with high potential of CERs synthesis regulation, since the most dramatic increase of CERs was observed in group of SK-MEL-5 cells treated with this phytocannabinoid. Nevertheless, it should be noted that since we have found no significant changes in CERs content in SK-MEL-5 cells after treatment with both CBD and CBG, we may suggest that synergistic effect of action of both examined phytocannabinoids may be more specific towards phospholipids than ceramides.

In present study, phosphatidylserine (PS) is the phospholipid class, which species’ content was not significantly affected by UVA radiation. This is important because the negative charge of the cell membrane depends on the presence of anionic phospholipid species, in particular PS, on the outer surface of the cell membrane. Revealed in our study the lack of quantitative differences with the simultaneous increase in the negative charge of membrane indicates changes in localization of PS on the external membrane surface that is confirmed by observed changes in the values of the negative zeta potential. The results of this study showed a significant increase in zeta potential in melanocytes after UVA irradiation, indicating PS externalization. Literature data show that in pathological conditions, clusters of melanosomes are secreted and apoptotic melanocytes are formed, with PS on the outer surface of the cell membrane. Macrophages then recognize PS on the outer surface of apoptotic cells, which triggers internalization of these cells. PS also undergoes translocation not only during apoptosis, but also in some physiological events related to intercellular signaling, such as exosomes. In addition, apoptotic melanocytes can also be incorporated into fibroblasts. This shows that PS-mediated endocytosis is commonly involved in many skin events^57^. In addition, PS exposure to the outer side of the cell membrane observed under oxidative stress induces immunosuppression that may promote the formation of metastasis^58^**,** it may be assumed that observed externalization of PS in UV-irradiated melanocytes could be associated with cancer development. Gangliosides, the amount of which increases in cancer cells, are also involved in changes in the charge on the membrane surface^59^. Surface exposure of PS has been reported in melanoma cells^60^, which seems paradoxical because PS acts as an apoptosis recognition signal for macrophages and cancer cells are able to evade apoptosis. Indeed it has been previously reported that melanoma, possibly by down-regulation of the host’s inflammatory immune responses, favor the establishment of metastasis in a PS-dependent manner^61^. Our study revealed also significant decrease of zeta potential in both UVA-irradiated and non-irradiated SK-MEL-5 cells after treatment with CBD and/or CBG, which indicate on the ability of examined phytocannabinoids to modulate location of PS on membranes of skin cancer cells. This effect is very important, as it was shown that externalization of PS on outer leaflet of cancer cells induces an immune-suppressive microenvironment to promote tumor growth, while blocking of PS externalization provides anti-tumor effects^62^. Moreover, our results confirmed that exposure of UVA-irradiated melanocytes to CBD and/or CBG action prevented UV-induced PS externalization. Considering the above it can be suggested that both phytocannabinoids may have anti-cancer potential preventing the development or progression of melanoma.

## Conclusion

In conclusion, based on obtained results it can be stated that UVA irradiation of melanocytes caused significant increase of PI and PC species content, which may indicate on metabolic changes in melanocytes towards the development of cancer that has been reported in previous studies. Our lipidomic study confirms that both phytocannabinoids CBD and CBG are able to regulate altered phospholipid metabolism in melanocytes as well as in SK-MEL-5 cells exposed to UVA. Treatment of UV-irradiated melanocytes with CBD led to significant reduction in the relative content of all statistically relevant phospholipid species. In contrast, the use of only CBG prevented the increase of only some PEo species and all relevant PC, SM and PI species. On the other hand, the use of both CBD and CBG almost completely prevented changes, induced by UVA, in the levels of PC, SM and PI, promoting at the same time further increase of PEo. Results obtained in the case of UVA-irradiated melanocytes treated with CBD and CBG are quite promising due to their ability to reverse pro-cancerogenic changes induced by UVA in phospholipid profile of these cells.

In the case of SK-MEL-5 cells, UVA caused dramatic up-regulation of all relevant phospholipid species, while exposure of these cells to both CBD and CBG resulted in a significant reduction in their relative content. It should be underlined that, the use of CBG alone also significantly prevented the UVA-induced increase in mentioned phospholipid species. In addition to that, both CBD and CBG have also possibility to modify SM-CER pathway in irradiated and non-irradiated SK-MEL-5 cells leading to different changes in relative contents of CER and SM species.

Our results revealed a different response of melanocytes and SK-MEL-5 cells to the action of phytocannabinoids, which clearly suggests their impact on different metabolic mechanisms in the case of physiological conditions and cancer. Our results also showed that the ability of the examined phytocannabinoids to reverse changes in the PL profile of UVA-irradiated SK-MEL-5 cells, which may lead to further tumor development, is limited.

According to our best knowledge here we present for the first time the effects of phytocannabinoids (CBD and CBG) action on melanocytes and melanoma cells exposed to UVA. Nevertheless, more detailed studies are needed to elucidate the exact molecular mechanisms responsible for the observed effects of these phytocannabinoids.

### Supplementary Information

Supplementary Information.

Supplementary Table S3.

Supplementary Table S4.

## Data Availability

The datasets generated during and/or analysed during the current study are available online as supplementary material.
