# Supplementary material for: Differences in the phospholipid profile of melanocytes and melanoma cells irradiated with UVA and treated with cannabigerol and cannabidiol
Source: Sci Rep. 2023 Sep 26;13:16121. doi: 10.1038/s41598-023-43363-9 (PMC10522606; doi:10.1038/s41598-023-43363-9)
Supplement: Supplementary file 1 — Supplementary Information. [file 41598_2023_43363_MOESM1_ESM.docx]

**Table S1.** Most abundant phospholipid species identified in non-irradiated (Melanocytes), irradiated with UVA (Melanocytes+UVA) and treated with cannabidiol [5 µM] (Melanocytes+CBD), cannabigerol [1 µM] (Melanocytes+CBG) and in combination (Melanocytes+CBD+CBG).

| **Phospholipid class** | **m/z** | **Retention time** | **Phospholipid specie** |
| --- | --- | --- | --- |
| **PC** | 818.5904 | 17.12 | PC(34:1) |
|  | 792.5746 | 17.39 | PC(32:0) |
|  | 950.6230 | 16.49 | PCp(46:11) |
|  | 840.5732 | 16.71 | PC(36:4) |
|  | 846.6213 | 16.92 | PC(36:1) |
|  | 844.6038 | 16.88 | PC(36:2) |
|  | 816.5748 | 17.13 | PC(34:2) |
|  | 820.6058 | 17.14 | PC(34:0) |
|  | 866.5908 | 16.53 | PC(38:5) |
|  | 842.5897 | 16.89 | PC(36:3) |
|  | 868.6052 | 16.39 | PC(38:4) |
|  | 870.6209 | 16.61 | PC(38:3) |
|  | 894.6222 | 16.40 | PC(40:5) |
|  | 874.5922 | 17.45 | PCp(40:7) |
|  | 892.6063 | 16.36 | PC(40:6) |
|  | 948.6066 | 16.58 | PCp(46:12) |
|  | 804.6101 | 17.42 | PCp(34:0) |
|  | 790.5585 | 17.34 | PC(32:1) |
|  | 826.5955 | 16.93 | PCp(36:3) |
|  | 900.6088 | 17.16 | PCp(42:8) |
|  | 848.6368 | 16.93 | PC(36:0) |
|  | 852.6101 | 16.75 | PCp(38:4) |
|  | 922.5938 | 16.61 | PCp(44:11) |
|  | 778.5957 | 17.58 | PCo(32:0) |
|  | 896.6372 | 16.36 | PC(40:4) |
|  | 854.6261 | 16.74 | PCp(38:3) |
|  | 954.7113 | 18.31 | PC(44:3) |
|  | 928.6940 | 18.12 | PC(42:2) |
|  | 864.5752 | 16.45 | PC(38:6) |
|  | 824.5792 | 16.68 | PCp(36:4) |
|  | 872.6366 | 16.74 | PC(38:2) |
|  | 890.5909 | 16.35 | PC(40:7) |
|  | 888.5753 | 16.27 | PC(40:8) |
|  | 806.6250 | 17.46 | PCo(34:0) |
|  | 976.6386 | 16.30 | PCp(48:12) |
|  | 830.6247 | 17.19 | PCp(36:1) |
|  | 764.5436 | 17.54 | PC(30:0) |
| **LPC** | 554.3463 | 20.37 | LPC(16:0) |
|  | 582.3771 | 20.10 | LPC(18:0) |
| **SM** | 761.5807 | 18.78 | SM(d34:1) |
|  | 857.6755 | 18.11 | SM(d41:2) |
|  | 845.6747 | 18.29 | SM(d40:1) |
|  | 817.6428 | 18.44 | SM(d38:1) |
|  | 789.6118 | 18.57 | SM(d36:1) |
|  | 763.5956 | 18.77 | SM(d34:0) |
|  | 869.6742 | 18.09 | SM(d42:3) |
|  | 843.6591 | 18.25 | SM(d40:2) |
|  | 759.5655 | 18.79 | SM(d34:2) |
|  | 787.5970 | 18.57 | SM(d36:2) |
|  | 847.6890 | 18.32 | SM(d40:0) |
|  | 871.6911 | 18.16 | SM(d42:2) |
|  | 791.6234 | 18.49 | SM(d36:0) |
| **LPE** | 480.3087 | 10.33 | LPE(18:0) |
|  | 500.2770 | 9.91 | LPE(20:4) |
|  | 452.2785 | 10.66 | LPE(16:0) |
|  | 478.2928 | 10.34 | LPE(18:1) |
|  | 526.2936 | 9.89 | LPE(22:5) |
| **PE** | 750.5420 | 6.95 | PEo(38:5) |
|  | 722.5125 | 7.14 | PEo(36:5) |
|  | 748.5279 | 7.05 | PEo(38:6) |
|  | 778.5724 | 6.86 | PEo(40:5) |
|  | 774.5421 | 6.83 | PEo(40:7) |
|  | 776.5575 | 6.94 | PEo(40:6) |
|  | 804.5903 | 7.01 | PEo(42:6) |
|  | 746.5117 | 7.01 | PEo(38:7) |
|  | 700.5277 | 7.43 | PEo(34:2) |
|  | 724.5257 | 7.22 | PEo(36:4) |
|  | 726.5414 | 7.33 | PEo(36:3) |
|  | 728.5587 | 7.34 | PEo(36:2) |
|  | 698.5122 | 7.45 | PEo(34:3) |
|  | 720.4954 | 7.16 | PEo(36:6) |
|  | 780.5898 | 6.92 | PEo(40:4) |
|  | 752.5585 | 6.75 | PEo(38:4) |
|  | 772.5262 | 6.84 | PEo(40:8) |
|  | 806.6053 | 6.74 | PEo(42:5) |
|  | 802.5745 | 6.74 | PEo(42:7) |
|  | 746.5685 | 7.31 | PEo(42:9) |
|  | 820.5273 | 6.28 | PEo(44:12) |
|  | 702.5432 | 7.52 | PEo(34:1) |
|  | 718.5350 | 7.50 | PE(34:0) |
|  | 754.5736 | 7.20 | PEp(36:6) |
|  | 832.6213 | 6.68 | PEo(44:6) |
|  | 730.5704 | 7.40 | PEo(36:1) |
| **PI** | 885.5472 | 3.83 | PI(38:4) |
|  | 887.5636 | 3.84 | PI(38:3) |
|  | 915.5954 | 3.47 | PI(40:3) |
|  | 921.6399 | 3.99 | PI(40:0) |
|  | 883.5332 | 3.84 | PI(38:5) |
|  | 861.5478 | 3.92 | PI(36:2) |
|  | 1031.7489 | 3.90 | PI(48:1) |
|  | 863.5651 | 3.96 | PI(36:1) |
|  | 857.5159 | 3.85 | PI(36:4) |
|  | 913.5801 | 3.76 | PI(40:4) |
|  | 911.5644 | 3.84 | PI(40:5) |
|  | 835.5323 | 3.99 | PI(34:1) |
|  | 859.5329 | 3.89 | PI(36:3) |
|  | 889.5766 | 3.89 | PI(38:2) |
|  | 917.6085 | 3.47 | PI(40:2) |
|  | 937.5803 | 3.56 | PI(42:6) |
|  | 971.6659 | 4.44 | PI(44:3) |
|  | 909.5490 | 3.84 | PI(40:6) |
|  | 1033.7629 | 3.90 | PI(48:0) |
|  | 907.5328 | 3.89 | PI(40:7) |
| **PS** | 810.5261 | 8.91 | PS(38:4) |
|  | 788.5435 | 9.34 | PS(36:1) |
|  | 832.5120 | 9.17 | PS(40:7) |
|  | 812.5425 | 9.09 | PS(38:3) |
|  | 834.5262 | 8.87 | PS(40:6) |
|  | 836.5426 | 9.03 | PS(40:5) |
|  | 786.5266 | 9.20 | PS(36:2) |
|  | 808.5111 | 9.33 | PS(38:5) |
|  | 838.5567 | 8.92 | PS(40:4) |
|  | 814.5575 | 9.34 | PS(38:2) |

**Table S2.** Most abundant phospholipid species identified in SK-MEL-5 cells, non-irradiated (SK-MEL-5) and irradiated with UVA (SK-MEL-5+UVA) and treated with cannabidiol [5 µM] (SK-MEL-5+CBD), cannabigerol [1 µM] (SK-MEL-5+CBG) and in combination (SK-MEL-5+CBD+CBG).

| **Phospholipid class** | **m/z** | **Retention time** | **Phospholipid specie** |
| --- | --- | --- | --- |
| **PC** | 868.6050 | 17.23 | PC(38:4) |
|  | 840.5744 | 17.58 | PC(36:4) |
|  | 818.5890 | 18.31 | PC(34:1) |
|  | 816.5765 | 18.35 | PC(34:2) |
|  | 844.6037 | 17.94 | PC(36:2) |
|  | 866.5915 | 17.31 | PC(38:5) |
|  | 868.5488 | 17.20 | PCp(40:10) |
|  | 864.5751 | 17.31 | PC(38:6) |
|  | 846.6237 | 17.95 | PC(36:1) |
|  | 892.6062 | 17.07 | PC(40:6) |
|  | 842.5901 | 17.86 | PC(36:3) |
|  | 870.6218 | 17.28 | PC(38:3) |
|  | 820.6079 | 18.41 | PC(34:0) |
|  | 792.5769 | 18.66 | PC(32:0) |
|  | 900.6699 | 17.04 | PC(40:2) |
|  | 790.5605 | 18.61 | PC(32:1) |
|  | 894.6218 | 17.09 | PC(40:5) |
|  | 872.6386 | 17.71 | PC(38:2) |
|  | 870.5662 | 17.30 | PCp(40:9) |
|  | 896.6386 | 17.47 | PC(40:4) |
|  | 898.5905 | 18.41 | PCp(42:9) |
|  | 900.6042 | 18.35 | PCp(42:8) |
|  | 926.6200 | 18.06 | PCp(44:9) |
| **LPC** | 554.3429 | 22.91 | LPC(16:0) |
|  | 578.3438 | 22.83 | LPC(18:2) |
|  | 582.3799 | 22.47 | LPC(18:0) |
|  | 580.3616 | 22.85 | LPC(18:1) |
|  | 602.3452 | 22.36 | LPC(20:4) |
|  | 552.3289 | 23.91 | LPC(16:1) |
| **PE** | 766.5379 | 7.50 | PE(38:4) |
|  | 714.5092 | 8.24 | PE(34:2) |
|  | 750.5427 | 7.51 | PEo(38:5) |
|  | 738.5122 | 7.67 | PE(36:4) |
|  | 744.5528 | 7.87 | PE(36:1) |
|  | 742.5387 | 7.88 | PE(36:2) |
|  | 702.5444 | 8.09 | PEo(34:1) |
|  | 764.5230 | 7.56 | PE(38:5) |
|  | 762.5081 | 7.53 | PE(38:6) |
|  | 752.5568 | 7.65 | PEo(38:4) |
|  | 716.5205 | 7.99 | PE(34:1) |
|  | 748.5251 | 7.50 | PEo(38:6) |
|  | 674.5125 | 8.12 | PEo(32:1) |
|  | 722.5124 | 7.62 | PEo(36:5) |
|  | 790.5409 | 7.38 | PE(40:6) |
|  | 766.4835 | 7.49 | PEp(40:10) |
|  | 740.5217 | 7.78 | PE(36:3) |
|  | 700.5274 | 8.08 | PEo(34:2) |
|  | 768.5505 | 7.55 | PE(38:3) |
|  | 774.5415 | 7.38 | PEo(40:7) |
|  | 776.5590 | 7.52 | PEo(40:6) |
|  | 778.5742 | 7.58 | PEo(40:5) |
|  | 728.5577 | 7.90 | PEo(36:2) |
|  | 792.5519 | 7.47 | PE(40:5) |
|  | 726.5409 | 8.12 | PEo(36:3) |
|  | 724.5253 | 7.98 | PEo(36:4) |
|  | 742.4818 | 8.03 | PEp(38:8) |
|  | 788.5226 | 7.89 | PE(40:7) |
|  | 780.5871 | 7.66 | PEo(40:4) |
|  | 794.5688 | 7.62 | PE(40:4) |
|  | 746.5114 | 7.63 | PEo(38:7) |
|  | 768.4974 | 7.67 | PEo(40:10) |
|  | 634.4447 | 8.33 | PE(28:0) |
|  | 736.4931 | 7.78 | PE(36:5) |
| **LPE** | 500.2809 | 10.20 | LPE(20:4) |
|  | 480.3086 | 10.45 | LPE(18:0) |
|  | 452.2792 | 10.74 | LPE(16:0) |
|  | 478.2954 | 10.61 | LPE(18:1) |
|  | 476.2781 | 10.75 | LPE(18:2) |
|  | 524.2808 | 10.15 | LPE(22:6) |
|  | 526.2904 | 10.17 | LPE(22:5) |
|  | 502.2910 | 10.85 | LPE(20:3) |
| **PI** | 885.5492 | 4.56 | PI(38:4) |
|  | 913.5769 | 3.76 | PI(40:4) |
|  | 943.6298 | 3.70 | PI(42:3) |
|  | 915.5964 | 3.99 | PI(40:3) |
|  | 881.5185 | 3.59 | PI(38:6) |
|  | 857.5163 | 4.50 | PI(36:4) |
|  | 945.6496 | 3.70 | PI(42:2) |
|  | 887.5654 | 3.75 | PI(38:3) |
|  | 859.5322 | 4.83 | PI(36:3) |
|  | 833.5189 | 4.84 | PI(34:2) |
|  | 883.5344 | 4.62 | PI(38:5) |
|  | 909.5498 | 4.49 | PI(40:6) |
|  | 889.5811 | 4.26 | PI(38:2) |
|  | 911.5631 | 4.75 | PI(40:5) |
|  | 865.5811 | 4.55 | PI(36:0) |
|  | 861.5498 | 4.01 | PI(36:2) |
|  | 879.5029 | 3.74 | PI(38:7) |
| **PS** | 868.6050 | 17.23 | PS(42:3) |
|  | 840.5744 | 17.58 | PS(40:3) |
|  | 816.5765 | 18.32 | PS(38:1) |
|  | 844.6037 | 17.94 | PS(40:1) |
|  | 818.5891 | 18.30 | PS(38:0) |
|  | 866.5915 | 17.31 | PS(42:4) |
|  | 864.5751 | 17.31 | PS(42:5) |
|  | 870.6218 | 17.28 | PS(42:2) |
|  | 892.6062 | 17.07 | PS(44:5) |
|  | 842.5901 | 17.86 | PS(40:2) |
|  | 846.6237 | 17.95 | PS(40:0) |
|  | 894.6218 | 17.09 | PS(44:4) |
|  | 790.5605 | 18.61 | PS(36:0) |
| **SM** | 761.5822 | 20.75 | SM(d34:1) |
|  | 871.6901 | 19.75 | SM(d42:2) |
|  | 869.6743 | 19.93 | SM(d42:3) |

**Table S3**. Peak area of each phospholipid species identified in non-irradiated melanocytes (Melanocytes), irradiated with UVA (Melanocytes+UVA) and treated with cannabidiol [5 µM] (Melanocytes+CBD), cannabigerol [1 µM] (Melanocytes+CBG) and in combination (Melanocytes+CBD+CBG). Provided as separated csv file.

**Table S4**. Peak area of each phospholipid species identified in SK-MEL-5 cells, non-irradiated (SK-MEL-5) and irradiated with UVA (SK-MEL-5+UVA) and treated with cannabidiol [5 µM] (SK-MEL-5+CBD), cannabigerol [1 µM] (SK-MEL-5+CBG) and in combination (SK-MEL-5+CBD+CBG). Provided as separated csv file.

**Table S5**. Most abundant ceramide species (CER[NS], CER[NDS]) identified in melanocytes and SK-MEL-5 cells examined in the study*.*

| **Ceramide class** | **m/z** | **Retention time** | **Ceramide specie** |
| --- | --- | --- | --- |
| CER[NS] | 650.6377 | 41.68384 | Cer(d18:1/24:0) |
|  | 622.608 | 36.79566 | Cer(d18:1/22:0) |
|  | 594.575 | 39.95251 | Cer(d18:1/20:0) |
|  | 536.4954 | 42.8122 | Cer(d18:2/16:0) |
|  | 676.652 | 43.48643 | Cer(d18:1/26:1) |
|  | 562.5127 | 43.93559 | Cer(d18:2/18:1) |
|  | 564.5269 | 41.84672 | Cer(d18:1/18:1) |
|  | 538.5112 | 44.1356 | Cer(d18:1/16:0) |
|  | 590.5441 | 41.35246 | Cer(d18:2/20:1) |
|  | 592.5573 | 44.47114 | Cer(d18:2/20:0) |
|  | 566.5417 | 44.27735 | Cer(d18:1/18:0) |
|  | 564.5318 | 35.01591 | Cer(d16:2/20:0) |
|  | 678.6656 | 43.17907 | Cer(d18:1/26:0) |
|  | 620.589 | 37.85562 | Cer(d18:2/22:0) |
|  | 550.5141 | 42.34468 | Cer(d15:2/20:0) |
|  | 648.6224 | 41.70807 | Cer(d18:1/24:1) |
| CER[NDS] | 652.6532 | 33.38246 | Cer(d18:0/24:0) |
|  | 624.623 | 34.20048 | Cer(d18:0/22:0) |
|  | 678.662 | 24.77687 | Cer(d18:0/26:1) |
|  | 540.524 | 29.30531 | Cer(d18:0/16:0) |
|  | 568.5581 | 33.74205 | Cer(d18:0/18:0) |
|  | 566.5417 | 33.72401 | Cer(d18:0/18:1) |
|  | 650.6379 | 33.26106 | Cer(d18:0/24:1) |
|  | 596.5908 | 31.68722 | Cer(d18:0/20:0) |


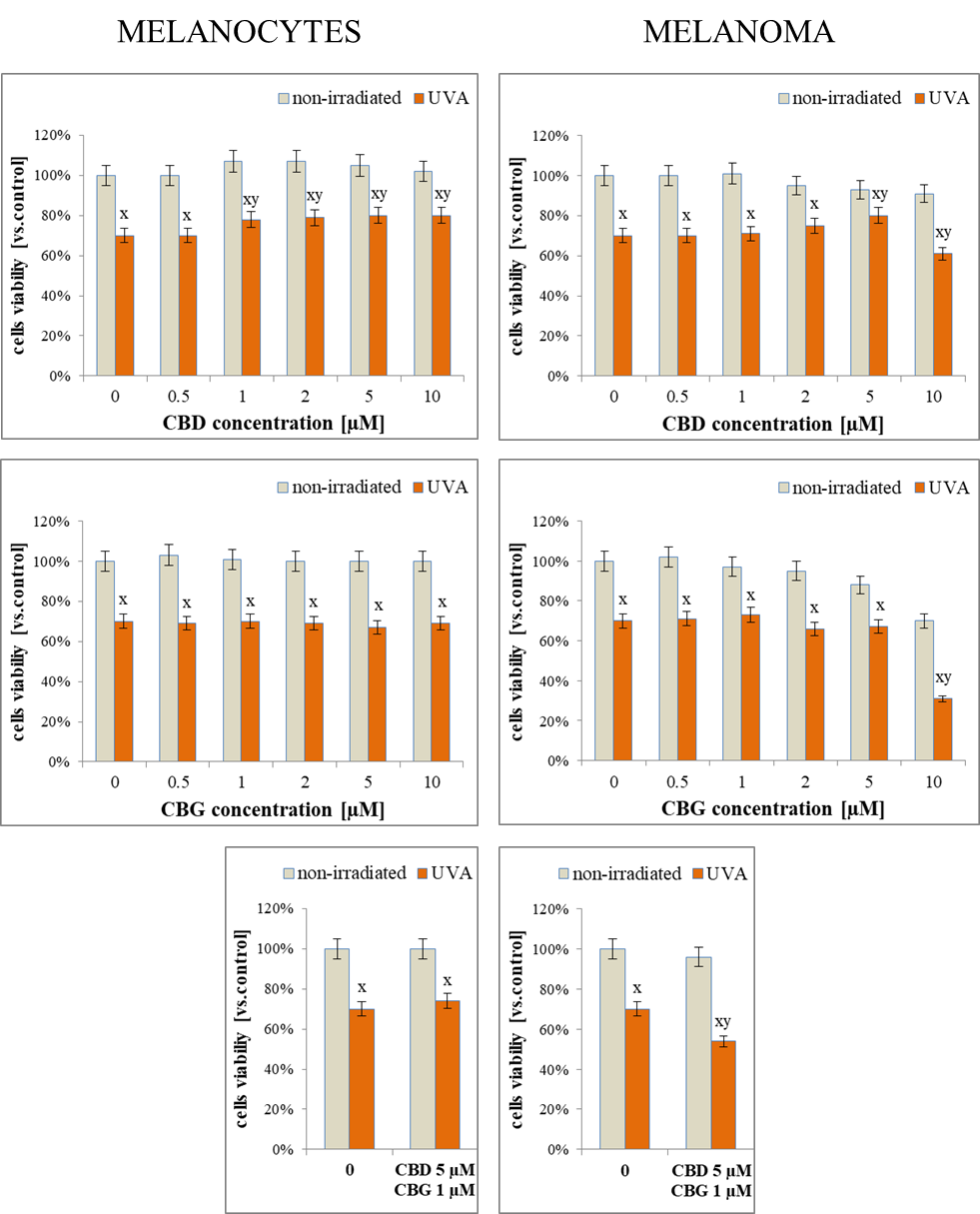


**Figure F1.** The effect of cannabidiol (CBD) and cannabigerol (CBG) used separate or combined on the viability of non-irradiated or UVA irradiated (18 J/cm^2^) melanocytes (ATCC PCS-200-012) and melanoma cells (ATCC HTB-70; SK-MEL-5) cultured *in vitro*. Data obtained by the MTT test (Fotakis & Timbrell, Toxicol. Lett., 2006: 160, 171–177). Mean values ± SD are presented. ^x^ statistically significant differences vs. non-irradiated cells, p < 0.05; ^y^ statistically significant differences vs. cells non-treated with CBD/CBG, p < 0.05.
